# Shear-wave elastography for assessment of trapezius muscle stiffness: Reliability and association with low-level muscle activity

**DOI:** 10.1371/journal.pone.0234359

**Published:** 2020-06-10

**Authors:** Žiga Kozinc, Nejc Šarabon

**Affiliations:** 1 University of Primorska, Faculty of Health Sciences, Polje, Izola, Slovenia; 2 University of Primorska, Andrej Marušič Institute, Muzejski trg, Koper, Slovenia; 3 S2P, Science to practice, Ltd., Laboratory for Motor Control and Motor Behavior, Tehnološki park, Ljubljana, Slovenia; 4 InnoRenew CoE, Human Health Department, Livade, Izola, Slovenia; University of Belgrade, SERBIA

## Abstract

**Purpose:**

Shear-wave elastography has been recognized a useful tool for quantifying muscle stiffness, commonly reported as shear modulus, however the reports on reliability are often limited to test-retest correlations. In this study, we explored the reliability of shear-wave elastography for assessment of the trapezius muscle stiffness and its relationship with low-level muscle activity.

**Methods:**

Twenty participants were included in a two-session experiment. Measurements of shear modulus and muscle activity were performed at rest and during low-level activity, induced by shoulder abduction without additional external resistance.

**Results:**

Good to excellent intra-session repeatability (ICC > 0.80) and moderate inter-rater and inter-session reproducibility (ICC = 0.66–0.74) were observed. Typical errors were acceptable (7.6% of the mean value) only for intra-session measurements in resting conditions, but not acceptable for all conditions with low-level muscle activity (10.2–16.6% of the mean value). Inverse relationships between shear modulus and muscle activity at 40° and 60° of shoulder abduction (r = -0.53 and -0.57) were observed on a group level. We also found higher shear modulus in males compared to females, for the parallel probe position compared to the perpendicular position (in relation to muscle fiber orientation), and for the dominant side of the body compared to the non-dominant side.

**Conclusions:**

This study showed an inverse relationship between muscle activity in low-level range and shear modulus on a group level, suggesting inherent passive stiffness could account for a larger portion of the variance (compared to muscle activity) in shear modulus when the muscle activity is low. Our results imply that shear-wave elastography can be used in research exploring muscle stiffness, however, caution is needed since only intra-session examination in resting conditions showed acceptable within-participant typical errors. The secondary analyses of the study showed higher shear modulus for males, for the non-dominant side of the body and for the parallel orientation of the ultrasound probe.

## Introduction

Shear-wave elastography (SWE) is a relatively novel ultrasound method for assessing mechanical properties of body tissues [1,2]. In addition to real-time imaging, modern ultrasound devices that utilize SWE technique enable quantification of tissue stiffness, expressed as shear modulus (kPa). In physical terms, stiffness is defined as the relationship between stress (e.g. force) and strain (deformation). A growing body of literature demonstrates the utility of SWE method for assessing muscle stiffness [3–12]. The shear modulus obtained with SWE has been validated for assessing muscle stiffnes, by using isolated meat speciments and comparing it to direct mechanical stiffness measures. The validity was confirmed throughout the full normal range of tension for appendicular skeletal muscle [13]. The reliability of the SWE for assessing muscle stiffness has been shown to be good or excellent for several muscle groups, including lower limb [5,11,12,14], trunk [4,10] and upper limb muscles [3,6–9]. It has been suggested that SWE could also be used to monitor muscle activity [9,12,15,16]. Namely, strong within-individual associations were shown between shear modulus values and muscle activity or muscle force [9,12,15].

The shear modulus has also been proved to be sensitive to the acute effects of muscle stretching [17–19] and warm-up [20]. Moreover, it is suggested that muscle stiffness is influenced by sex and age [21,22]. Both lower (gastrocnemius, rectus femoris muscles), similar (soleus muscles) [21] and higher (biceps brachii) [22] shear modulus values were found in elderly, compared to young participants. Studies including gender comparison in terms of shear modulus values are inconclusive and the observed differences are usually small [22]. Overall, the previous findings tend to show slightly higher values in females [22,23]. One study also suggested an interaction between sex and age, with similar values between genders in young age and increasingly higher differences (higher values in females) with increasing age [22]. Finally, increased shear modulus values for muscles were shown by the SWE method in populations with neuromuscular diseases, such as cerebral palsy [24] and Parkinson’s disease [25]. Some methodological concerns for shear modulus assessment should be acknowledged. The choice of body site to conduct a measurement may be an important factor of shear modulus score. For instance, higher shear modulus values were reported on the dominant side, compared to non-dominant side for the trapezius muscle [6]. On the other hand, studies exploring lower limb muscles have not reported such differences [12,14]. Finally, several studies have highlighted the importance of the consistency of the probe orientation, as the shear modulus may substantially decrease in a position perpendicular to the muscle fibers, compared to positioning parallel with the muscle fibers [8,26].

The trapezius muscle, particularly its upper portion, has been subjected to extensive research in view of its potential role in developing chronic upper back and neck syndromes [27–34]. During a 30-min writing task, the activity of the upper trapezius was reported to be fourfold higher in patients with chronic neck pain, compared to healthy controls [31]. One study found no differences in trapezius shear modulus between patients with chronic neck pain and controls [28]. Determining the limits of trapezius activity that represent an increased risk for developing neck syndromes is difficult since most studies used normalizations to non-standard reference tasks [27,29,30] instead of maximal voluntary contraction (MVC) tasks. Cui et al. [32] reported the activity of upper trapezius to be 5–7% MVC during typing, with little difference between sitting and standing posture. In another study, a wide range of average activity of the trapezius during 30-min office (0.5–9.3% MVC) or light manual work (0.6–12.5% MVC) was reported, with no difference between the work types [33]. Previous research has also suggested differences in trapezius muscle stiffness (assessed by SWE) between the dominant and non-dominant side of the body during rest [6], while no differences in upper trapezius activity between body sides were recorded during computer-based tasks [34].

The reliability of SWE for assessing muscle stiffness of the trapezius has been consistently reported as good to excellent [6–9]. However, some of these studies did not investigate inter-rater [6,8] or inter-session [7,9] reproducibility, and only one study reported typical errors and minimal detectable changes [6]. Since test-retest correlations are dependent upon sample heterogeneity, reporting only intra-class correlation coefficient can be somewhat misleading [35]. Typical errors and minimal detectable changes should be calculated and reported as well in order to present a comprehensive overview of reliability. While the association between muscle activity and muscle stiffness has been consistently reported on other muscles [9,12,15,16], these studies used a large range of contraction intensities. Potential associations between smaller changes in muscle activity (such as typically seen in trapezius muscle during office or light manual work [32,33]) and changes in shear modulus are yet to be explored. Notably, all of the aforementioned studies examined the within-individual relationship between shear modulus and muscle activity. It remains to be explored whether there are associations between shear modulus and muscle activity at different levels of contractions on a group level as well (i.e. associations between shear modulus and muscle activity across a sample at a single muscle activity level). Knowledge of the relationship on a group level would enable between participant comparisons in terms of muscle activity by using SWE. Establishing the clear relationships between shear modulus and muscle activity on both individual and group level could represent an important methodological advantage, as SWE method could represent an easy-to-use and non-invasive tool to monitor the risk for development of neck discomfort or pain syndromes that are associated with muscle activity level [36].

In view of the outlined gaps in the literature, the primary aim of this study was to determine the reliability of SWE for assessing trapezius muscle stiffness at rest and low-level activation, and to assess the relationship between trapezius muscle stiffness and muscle activity on an individual level and on a group level. For this purpose, muscle stiffness (i.e. shear modulus) and muscle activity for upper trapezius muscle were assessed in resting position and during low-level activity (shoulder abduction with no additional resistance) conditions. The secondary aim of the study was to assess the difference in muscle stiffness measures between genders, between the dominant and non-dominant side of the body and between parallel and perpendicular positioning of the ultrasound probe. We hypothesized that the repeatability and reproducibility of the shear modulus measurements will be excellent in resting conditions, and acceptable in conditions with low-level muscle activation. Furthermore, we hypothesized that our results will confirm the positive within-individual associations between shear modulus and muscle activity, and that these associations will also be shown on a group level. Finally, we expected to observe slightly higher shear modulus in females compared to males, higher values on the dominant side of the body compared to non-dominant side, and higher values with parallel positioning of the ultrasound probe, compared to the perpendicular position.

## Methods

### Participants

**Fig 1:**
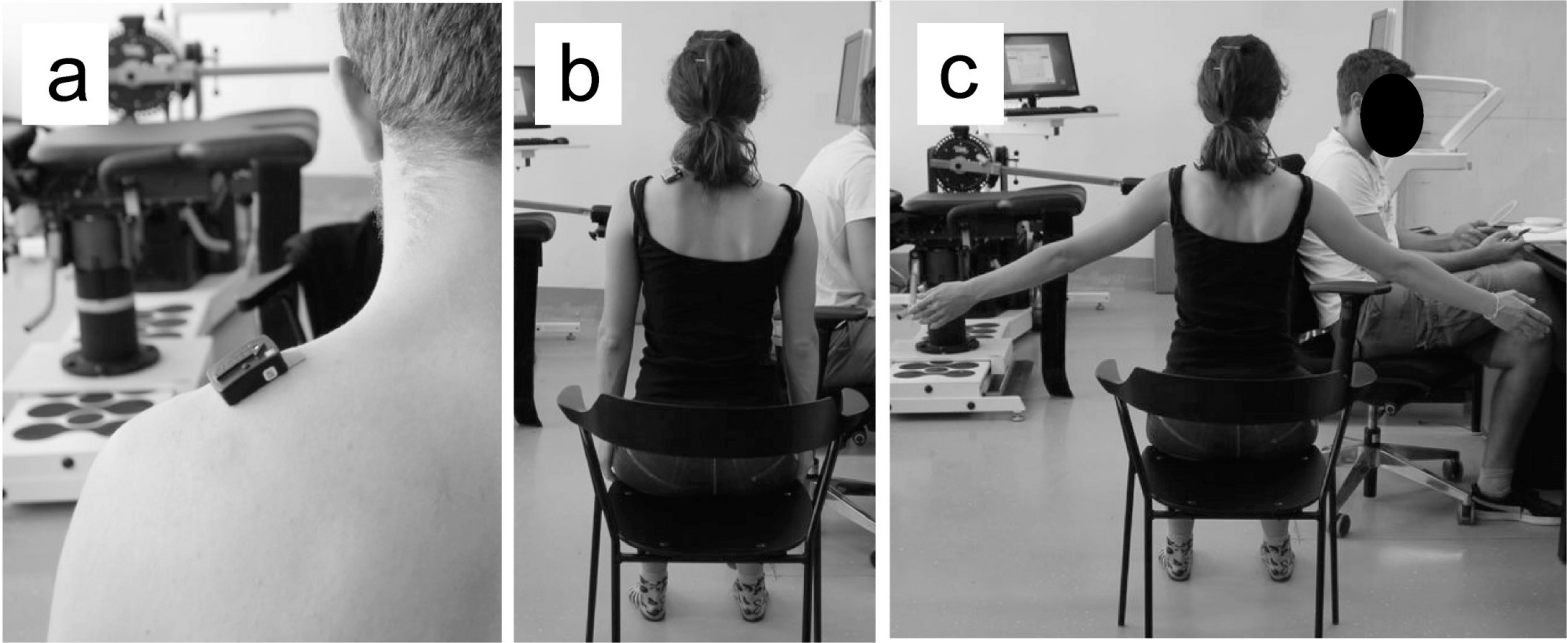
Muscle activity and shear modulus were recorded in the same conditions, with the location of the electrode and the ultrasound probe at 50% of the line from the acromion to the spine of C7 (A). Measurements were performed in a relaxed position (B) and with shoulder abduction of 40° and 60° (C).

**Fig 2:**
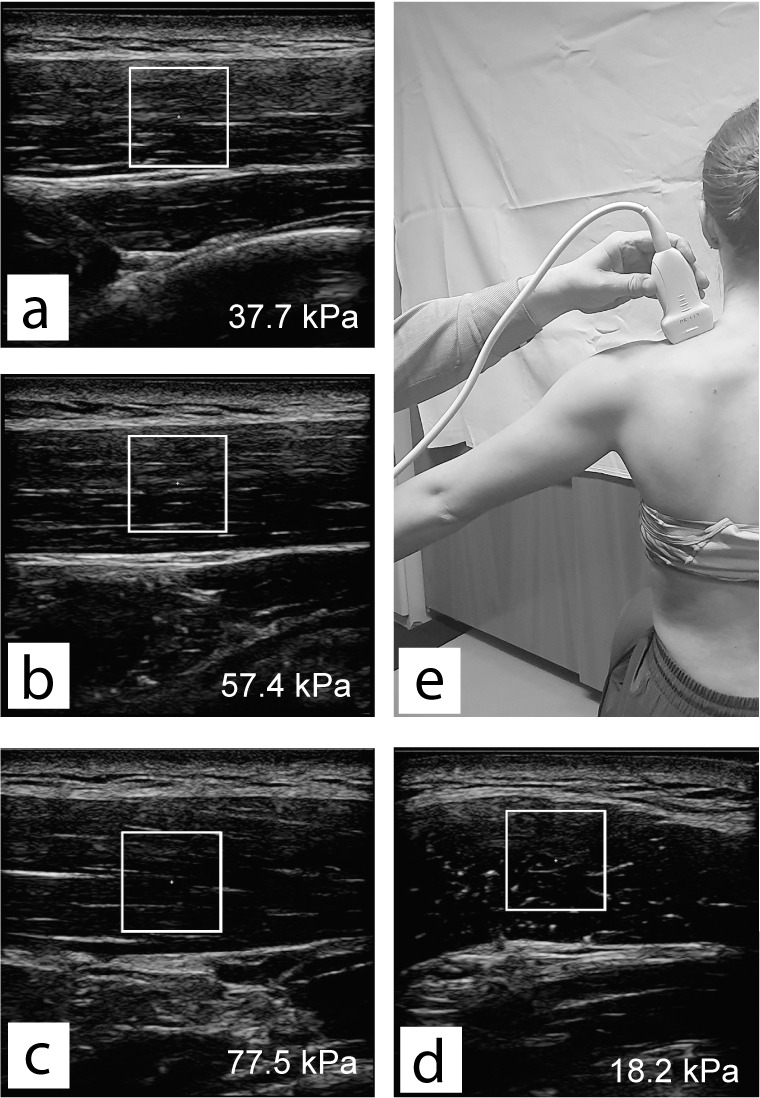
Examples of shear-wave scans in relaxed position (a), 40° of shoulder abduction (b), 60° of shoulder abduction (c) and for relaxed position with transverse probe positioning (d). Example of probe positioning on the subject is shown for the condition with 60°of shoulder abduction (e).

The study sample comprised of 20 healthy adults (8 males, 12 females; age: 31.4 ± 9.8 years; height: 171.7 ± 10.4 cm; body mass: 69.0 ± 12.8 kg). Based on the criteria of moderate association (r = 0.7), statistical power of 0.80 and alpha error of 0.05, the minimum sample size was 19 participants based on an a priori estimation. The inclusion criteria were age > 18 years and good health. The exclusion criteria were the presence of musculoskeletal injuries in the last 12 months and any history of shoulder girdle injuries and pain syndromes, presence of any of the non-communicable chronic diseases or any neuromuscular problems. Before the measurements, each participant was thoroughly informed about the aims and procedures of the study and signed an informed consent form prior to participation. The study was approved by the National Medical Ethics Committee of Slovenia (approval number 0120-690/2017/8) and was conducted in accordance with the Declaration of Helsinki. The individuals pictured in Fig 1 and Fig 2 have provided written informed consent to publish their image alongside the manuscript. They have given written informed consent (as outlined in PLOS consent form) to publish these case details.

### Study design and procedures

The measurements were conducted in two sessions that were 7–10 days apart. In the first session, the procedures were explained to the participants and informed consent was obtained. The experiment consisted of muscle stiffness assessment and muscle activity assessment. The order of these two sections was randomized between participants. The location of the muscle stiffness assessment, as well as the EMG electrode placement, was set at 50% of the line from the acromion to the spine of C7 (Fig 1A), which is in accordance with the SENIAM recommendations [37]. The electrode placement was conducted by the examiner, who was experienced with EMG measurements and did not conduct muscle stiffness assessments. The two examiners that performed the measurements were kinesiologists with sufficient knowledge of shoulder girdle anatomy, who had received training sessions (2 sessions lasting ~ 1 hour) by an expert in ultrasound imaging. These sessions included training of the probe positioning and discerning the different anatomical structures. Both examiners had at least a master’s degree in kinesiology and were familiar with the anatomical details of the musculoskeletal system of the upper body. The participants were required to attend both sessions at the same time of day. The examination room was air-conditioned to ensure the constant temperature of 23°C.

The shear modulus of the trapezius muscles was measured in three conditions: relaxed sitting posture (Fig 1B), and sitting with shoulder abducted to 40° and 60° (Fig 1C) (neutral position = 0°). Three repetitions with 1-min breaks in-between were performed in each condition to assess intra-session repeatability. The order of the conditions was randomized between participants, but was kept the same within the participant. In resting condition, the participants’ arms were relaxed and the hands were resting on his/her thighrs. The participants were instructed to completely relax for these measurements. For the low-level activity conditions, the level of abduction was determined with a goniometer. The axis of the goniometer was aligned with the estimated center of the humeral head (1–2 cm below the acromion), the stationary arm was aligned with the sternum, and the moving arm was aligned with the midline of the humerus [38]. The goniometer was operated by an examiner who was not performing shear modulus measurements at any time. Once the correct position was adopted, the goniometer was removed and the examiner began with the muscle stiffness assessment (~10 s). Participants were instructed to maintain the same position until the end of the measurement. One examiner performed all the measurements (i.e. three repetitions in all three conditions). To assess inter-rater reproducibility, a second rater performed additional three measurements in the condition with 40° of shoulder abduction during the first session. The second rater was blinded to the results of the first rater. We chose to assess inter-rater reproducibility only in one condition to reduce the number of trials and therby reduce the possible effects of fatigue. To assess inter-session reproducibility, the first examiner repeated the measurements during the second session. The break between repetitions lasted for 1 min and the break between conditions for 2 min. The first rater also performed additional three repetitions on the opposite side of the body (during the first session) and three repetitions with perpendicular probe positioning (during the second session), both in resting conditions.

Trapezius muscle activity was examined in the same three conditions as muscle stiffness. Three repetitions were performed for each condition (0°, 40° and 60° of shoulder abduction), and the measurements were repeated during the second session. The conditions were assumed in the same manner as for muscle stiffness assessment. Muscle activity recording began after the required arm position was adopted and the goniometer was removed. The duration of each repetition was set at 30 s. Similar as for muscle stiffness assessments, the break between repetitions was 1 min and the break between conditions was 2 min. For the purposes of muscle activity normalization, MVC task was performed. Participants were seated, abducted the shoulder to 40° and were instructed to resist the examiner, who provided manual resistance in direction of shoulder adduction at the elbow level (static conditions).

### Equipment and data processing

For the assessment of shear modulus, we used the Resona 7 ultrasound system (Mindray, Shenzhen, China). The ultrasound system was set to musculoskeletal SWE mode, which assumes the tissue density of 1000 kg/m3. Middle-sized linear probe (Model L11-3U, Mindray, Shenzhen, China) and water-soluble, hypoallergenic ultrasound gel (AquaUltra Basic, Ultragel, Budapest, Hungary) were used. The region of interest was fixed at 1 cm^2^ square shape. The depth was left to the choice of each examiner. Shear modulus data was transcribed into pre-prepared sheets immediately after the acquisition. The outcome measure for each condition was the mean value of eight quick consecutive scans, which is the maximum storage capacity of the device. Examples of shear wave scans with regions of interest and respective shear modulus values are depicted on Fig 2.

For EMG activity assessment, Trigno Delsys Wireless System was used (Delsys Inc., Massachusetts, USA). Pre-amplified wireless electrodes with self-adhesive stickers were placed on the upper portion of the trapezius muscle on the non-dominant side of the body. Prior to the electrode placement, the skin was shaved, slightly abraded (impedance < 5kΩ) and cleaned with alcohol. EMG data was processed with automatized custom-made Matlab routine (MATLAB R2018a, The MathWorks, Natick, USA). The signals were band pass filtered (20–500 Hz, second order) and rectified using root mean square function with 500 ms overlap. For each repetition, the outcome measure of interest was the average level of muscle activity over a time window from 5 to 25 s. The values were normalized to the maximal activity obtained during MVC trials, which were processed in the same manner.

### Statistical analysis

Statistical analysis was done in SPSS (version 20.0, SPSS Inc., Chicago, USA). Descriptive statistics were calculated and reported as mean ± standard deviation. Normality of the data distribution was checked with the Shapiro-Wilk test. Repeatability and reproducibility were assessed using two-way mixed single score (3,1) intra-class correlation coefficients (ICC). ICC scores were interpreted as fair (ICC 0.40–0.59), moderate (ICC 0.60–0.74) and good to excellent (ICC 0.75–1.00) [39]. For the intra-rater and inter-session repeatability ICC, data of only one examiner was used. Within-subject error was assessed using typical error and coefficients of variation (CV% = TE/mean × 100), as recommended by Hopkins (2000) [35]. The acceptable level of within-subject error was condsider at CV > 10%. Minimal detectable change was calculated (minimal detectable change = TE × 1.96 × √2) to determine the magnitude of change in shear modulus score that would exceed the threshold of measurement error at the 95% confidence level. To check for a systematic error between the trials, repeated measures analysis of variance was used. Pearson correlation coefficients were calculated to assess the associations between shear modulus scores and muscle activity (0.0–0.1 (no association), 0.1–0.4 (weak), 0.4–0.6 (moderate), 0.6–0.8 (strong) and >0.8 (very strong)) [40]. For the assessments on an individual level, the mean of the three shear modulus values (for each shoulder abduction level) obtained by the first rater were correlated with the mean of the corresponding EMG recordings. Associations on a group level were performed for each shoulder position separately, by correlating the mean of the three shear modulus scores for each position and the mean of the corresponding EMG recordings. Note that using the data from the second rater or data obtained on the second visit had no significant effect on the outcomes (difference in r = 0.01–0.02). The paired-sample t-test was used to evaluate the differences between shear modulus scores between sexes, body sides and probe orientations. The level of significance was set to p < 0.05 for all analyses.

## Results

### Reliability

The outcomes pertaining to reliability of shear modulus assessment and muscle activity recordings are presented in Table 1. Shear modulus values had good to excellent intra-session repeatability according to ICC (0.82–0.91), and moderate inter-rater (ICC = 0.74) and inter-session (ICC = 0.66–0.71) reproducibility. On the other hand, shear modulus coefficient of variations showed a high level of within-participant error for all conditions and comparisons (CV = 10.2–16.6%), except for intra-session assessment in 0° of shoulder abduction (CV = 7.6%). There were no systematic errors for shear modulus detected in any of the positions (p = 0.077-0.732). Muscle activity recordings had excellent intra-session repeatability (ICC = 0.96–0.99), with low within-participant error (CV = 7.0–8.0%) and no systematic errors among trials (p = 0.254-0.874). On the other hand, inter-session reproducibility for muscle activity was fair to moderate (ICC = 0.52–0.73), with very high within-participant errors (CV = 28.0–55.7%), but no systematic errors among trials (p = 0.156-0.718).

**Table 1:** Reliability of the shear modulus and muscle activity measurements.

Outcome	Shoulder position and comparison type	Trial 1	Trial 2	Trial 3	Reliability				Systematic error		Minimal detectable change
		M ± SD	M ± SD	M ± SD	ICC _(3,1)_	ICC 95% CI	TE (95% CI)	CV%	F	p	
Shear modulus (kPa)	Intra-session 0°	43.3 ± 11.8	45.3 ± 14.7	42.6 ± 12.7	0.91	0.81–0.96	3.3 (2.9–4.6)	7.6	2.68	0.081	9.3
	Intra-session 40°	59.6 ± 16	61.2 ± 19.2	61.1 ± 18.4	0.82	0.66–0.92	6.2 (4.9–8.4)	10.2	0.32	0.732	17.1
	Intra-session 60°	58.3 ± 20.1	54.9 ± 20.2	53.8 ± 17.7	0.84	0.69–0.93	7.3 (5.9–9.6)	13.1	2.73	0.077	20.2
	Inter-rater 40°	60.5 ± 16.5	55.6 ± 18.6	/	0.74	0.45–0.89	9.6 (5.8–13.9)	16.6	3.25	0.087	26.7
	Inter-session 0°	43.8 ± 12.8	46.7 ± 11.5	/	0.66	0.32–0.84	7.1 (3.4–11.6)	15.7	1.69	0.209	19.7
	Inter-session 40°	60.5 ± 16.5	64.6 ± 13.8	/	0.71	0.40–0.87	8.1 (4.6–12.3)	13.0	2.53	0.128	22.5
	Inter-session 60°	65.8 ± 19.4	69.8 ± 16.6	/	0.71	0.41–0.87	9.6 (5.5–14.4)	14.2	1.75	0.202	26.6
Muscle activity (%MVC)	Intra-session 0°	3.5 ± 3.2	3.7 ± 3.3	3.8 ± 3.5	0.96	0.91–0.98	0.3 (0.2–1.3)	7.9	1.42	0.254	0.8
	Intra-session 40°	10 ± 6.3	10.4 ± 7.5	10.2 ± 7.3	0.97	0.94–0.99	0.8 (0.6–1.8)	8.0	0.14	0.874	2.3
	Intra-session 60°	13.1 ± 9.2	13.3 ± 9.7	13.2 ± 8.8	0.99	0.97–0.99	0.9 (0.8–1.9)	7.0	0.31	0.738	2.6
	Inter-session 0°	3.7 ± 3.3	4.6 ± 3.5	/	0.52	0.12–0.77	2.3 (0.6–5.2)	55.7	1.61	0.221	6.4
	Inter-session 40°	10.1 ± 6.9	11.5 ± 5.2	/	0.73	0.44–0.88	3.0 (1.8–5.1)	28.0	2.17	0.156	8.4
	Inter-session 60°	13.3 ± 9	13.8 ± 5.4	/	0.67	0.33–0.85	4.4 (2.2–7.4)	32.4	0.13	0.718	12.2

M–mean; SD–standard deviation; MVC–maximal voluntary contraction; ICC _(3,1)_—two-way mixed, single score intra-class correlation coefficient; CI–confidence interval; TE–typical error; CV–coefficient of variation; F–F-test statistics for ANOVA; p – probability of alpha error for ANOVA.

### Relationship between shear modulus values and muscle activity

Within-participant associations between shear modulus and muscle activity were positive, very strong and consistent (r = 0.95 ± 0.06; range: 0.82–0.99). However, when relationships between shear modulus and muscle activity were evaluated between participants (i.e. on a group level), using data from each condition separately, we found moderate to strong inverse relationships at 40° of shoulder abduction (r = -0.53; p = 0.010) and at 60° of shoulder abduction (r = -0.67; p = 0.002), but no statistically significant relationship at rest (r = 0.43; p = 0.07). There was also a moderate positive association (r = 0.44; p = 0.048) between relative changes in shear modulus between 0° and 60° and relative change in muscle activity between the same positions. Relationships between shear modulus values were weak and not statistically significant between 0° and 60° conditions (r = 0.33; p = 0.141), moderate between 0° and 40° (r = 0.44; p = 0.041) and strong between 40° and 60° (r = 0.73; p < 0.002).

### Sex differences in muscle stiffness and muscle activity

Female participants had lower shear modulus (p = 0.017-0.043) compared to male participants in all three conditions (mean difference = 11.5 ± 5.3 kPa for 0° of shoulder abduction, 15.41 ± 6.8 kPa for 40° of shoulder abduction and 20.3 ± 7.7 kPa for 60° of shoulder abduction). There were no effects of sex on average muscle activity in any of the positions.

### Effects of body side and probe positioning

Shear modulus was statistically significantly higher (p = 0.016) on the non-dominant side of the body (43.7 ± 12.8 kPa), compared to the dominant side (37.8 ± 8.6 kPa). Positioning the probe perpendicular to muscle fibers orientation resulted in lower (p < 0.001) shear modulus (32.6 ± 7.4 kPa), compared to parallel orientation (46.7 ± 11.5 kPa).

## Discussion

The primary purpose of this study was to assess the reliability of SWE for quantifying trapezius muscle stiffness and to explore the relationship between muscle stiffness (i.e. shear modulus) and muscle activity. The secondary aim was to compare the shear modulus values between sexes, between dominant and non-dominant body sides, and between measurements with parallel and perpendicular positioning of the probe. We found good to excellent intra-rater repeatability and moderate inter-rater and inter-session reliability for shear modulus scores. Measurements of muscle activity with electromyography had excellent intra-session repeatability, but only fair to moderate inter-session reproducibility. Of note, within-individual errors for shear modulus measurements were high in general (CV > 10%), except for the relaxed conditions. In conditions of low-level activity (i.e. 5–15% MVC), positive within-participant and negative (i.e. inverse) between-participant relationships between shear modulus and muscle activity were found. Relative change in shear modulus correlated positively with the relative change in muscle activity. We observed higher shear modulus for males, for the non-dominant side of the body and for the parallel orientation of the ultrasound probe.

This study confirmed good to excellent intra-session repeatability and moderate inter-rater and inter-session reproducibility for assessment of muscle stiffness, quantified by shear modulus values obtained with SWE imaging method. Several previous studies have reported similar findings for the trapezius muscle [6–9], however, within-participant typical errors were rarely reported [6]. In a study by Xie et al. [6] very similar intra-session typical errors were found compared to the present study (CV = 6–8%) for trapezius shear modulus in resting position. Based on our results, the assessment of shear modulus is reliable in conditions of rest and low-level activity on a group level. This is also supported by the absence of systematic errors in all conditions. However, only assessment in the resting condition produced acceptable within-individual errors (CV < 10%). In summary, it seems that SWE is a reliable tool for assessing trapezius muscle stiffness, however, a high possibility of error needs to be considered when the measurements are conducted on an individual level.

Previous studies have demonstrated a strong positive linear relationship between shear modulus and muscle activity or muscle force [9,12,15,16]. Therefore, changes in muscle activity can be potentially monitored with SWE method. Previous studies used a broad range of contraction intensities and explored only within-participant associations. The present study involved relatively low levels of activity (10–15% MVC) and explored the relationship between shear modulus and muscle activity on an individual and also on a group level. As in the abovementioned studies, very strong relationships between shear modulus and muscle activity were found on an individual level. However, when examining between-participant associations, we observed moderate to strong inverse relationships between shear modulus and muscle activity in both conditions that induced low-level activity (40° and 60° of shoulder abduction), but not at rest. To the best of our knowledge, such evaluation has not been performed in previous studies. Our findings suggest that individuals with stiffer trapezius muscles showed lower activity levels when they abducted the shoulder to 40° and 60°. At first glance, this is a somewhat surprising finding in light of the abovementioned reports on the positive linear relationship between shear modulus and muscle activity on an individual level. However, it is reasonable to assume that participants with lower muscle stiffness need to activate a muscle to a greater extent to resist comparable external force. In biomechanical terms, stiffness is defined as the relationship between stress (e.g. force) and strain (deformation) [2]. Participants with lower mechanical muscle stiffness of the trapezius (and possibly other muscles, involved in shoulder abduction) are therefore expected to need higher levels of muscle activation to resist the external force (adduction torque, caused by the mass of the arms). However, additional activation would expectedly increase the stiffness [9,12,15,16]. We could speculate that baseline passive stiffness in the resting condition explains a greater proportion of the variance in shear modulus measured at low-levels of muscle activity. Once the activity levels increase, the activity level itself explains the larger proportion of the variance in the shear modulus. This hypothesis needs further research, as these assumptions are only partially supported by correlations between resting shear modulus and shear modulus at low-levels of activity (r = 0.33–0.44).

Previous studies have shown small differences in shear modulus between genders, with slight trend towards higher values for females [22,23]. In contrast, we found higher values for males. This suggest that gender effect on shear modulus could be muscle specific. On the other hand, the sample size was somewhat small for independent comparison of the means, and the number of males was lower than females (8 vs. 12). Further studies are clearly needed to establish the effect of gender on shear modulus. Interestingly, the shear modulus on the non-dominant side of the body was higher (43.7 ± 12.8 kPa) compared to the dominant side (37.8 ± 8.6 kPa). This is in contrast with a previous study, which reported higher muscle stiffness on the dominant side for trapezius [6]. However, the difference in that study was detected on the posterior side of the trapezius. Previous studies reported no difference between body sides for hamstring [12] and quadriceps [14] shear modulus values. Therefore, it remains unclear whether shear modulus values are sensitive to changes induced by the preferential utility of muscle on the (non)dominant side. Future experiments should include measurements on both sides of the body when possible, in order to provide more information regarding differences between dominant and non-dominant sides in view of muscle stiffness scores. Positioning the probe perpendicular to muscle fibers orientation resulted in lower shear modulus (32.6 ± 7.4 kPa), compared to the parallel probe orientation (46.7 ± 11.5 kPa), which is in accordance with previous reports [8,26]. This highlights the importance for standardizing probe orientation and potentially adjusting it in case of investigating active muscles at different contraction intensities that possibly induce a change in muscle fibers orientation and superficial muscle geometry in general.

### Limitations

The limitations of the study need to be acknowledged. First, the examiners in our study were not trained radiologists or surgeons, but had sufficient anatomy knowledge and received training from an expert radiologist. Non-expert examiners were employed due to the claim that SWE imaging is not as operator-dependent as other ultrasound-based methods [2]. However, it cannot be excluded that higher reliability scores could be obtained if an expert radiologist performed the measurements. Future studies should directly compare the reliability scores between examiners of different skill levels to clarify this concern. The position of the shoulder in the present study was carefully determined, but was monitored only visually. Therefore, small deviations from the initial position during the trials cannot be excluded. Lastly, fatigue could have affected the shear modulus and muscle activity measurements. We aimed to minimize such effects with sufficient breaks and randomization of the conditions, however, a low level of fatigue cannot be excluded despite low-levels of muscle activity.

## Conclusion

We found good to excellent intra-session repeatability and moderate inter-rater and inter-session reproducibility, with no systematic errors. While this implies that SWE can be used in research exploring muscle stiffness, caution is warranted in clinical settings since only intra-session examination in resting conditions showed acceptable errors for individual participants. Shear modulus values increased in parallel with muscle activity for each participant. Within the conditions of low-level activity, the shear modulus values were inversely related to muscle activity on a group level. We also confirmed differences in muscle stiffness between sexes, with higher values in male participants, probe orientation, with higher values for parallel orientation, and body side, with higher values for non-dominant side. These factors should be considered and respected in research and clinical settings.

## Abbreviation list

CV: coefficient of variation
ICC: intra-class correlation coefficient
MVC: maximal voluntary contraction
SWE: Shear-wave elastography
